# Effects of different levels of vitamin premix in finisher diets on performance, immuno-competence and meat lipid oxidation of chickens fed on corn-soybean meal

**Published:** 2013

**Authors:** Hoseein Moravej, Majid Alahyari-Shahrasb, Ali Kiani, Mona Bagherirad, Mahmood Shivazad

**Affiliations:** 1 *Department of Animal Science, Faculty of Agriculture and Natural Resources, University of Tehran, Karaj, Iran; *; 2 *Department of Animal and Poultry Science, College of Aburaihan, University of Tehran, Pakdasht, Iran.*

**Keywords:** Broiler, Immunocompetence, Lipid peroxidation, Vitamin premix

## Abstract

The present study was carried out to examine the effects of a vitamin premix (VP) reduction or withdrawal from finisher diet (29-43 days) on performance, immuno-competence, and characteristics of leg bones and meat lipid oxidation of chickens fed on corn-soybean meal based diet. A total of 900 male broiler chickens (Ross 308) were allocated to five treatment groups (0, 33%, 66%, 100% and 133% VP), with nine replicates per treatment group. At 29 and 36 days of ages, four birds from each replicate were injected with sheep red blood cells (SRBC). The cell-mediated immunity was determined via phytohemagglutinin (PHA) and 1-chloro 2-4-dinitrobenzen (DNCB) at 34 and 42 days of ages. At 33, 38 and 43 days of age, 42 days of ages, and two birds of each replicate were slaughtered and bone parameters measured. The oxidative stability was evaluated by thiobarbituric acid reactive substances (TBARS) on the thigh samples that were stored for 90 day at -80 ˚C. The results showed that reduction or withdrawal of VP from diets at different time points of the finisher period did not affect performance, immunocompetence and characteristics of leg bones. Results of TBARS showed that lipid peroxidation of the treatment without VP was significantly higher than of the other treatments when slaughtered at 43 days of age. Finally, the results of this study demonstrated that it is not possible to reduce the VP in finisher broilers’ diets without negative effects on meat quality during the time of freezing.

## Introduction

Maiorka *et al.* indicated that vitamin premix (VP) withdrawal at 42 day of age did not impair feed intake or weight gain, but significantly affected feed conversion ratio of broiler chickens.^1^ In contrast, Khajali *et al.* reported that removing vitamin and trace mineral premixes from broiler diets from 28 to 49 days of age had little impact on growth performance.^2^ Vitamin and trace mineral deficiencies have been shown to suppress immunocompetence.^2^ Therefore, the response of the immune system needs to be considered. Deyhim and Teeter showed that removal of the VP from broiler rations did not affect immunological competence as judged by antibody titre to sheep erythrocyte injection.^3^ Khajali *et al.* suggested that the vitamin and trace mineral contents of the finisher diet were sufficiently high to maintain a humoral immune response.^2^

Duration of removal period, different levels of VP, meat quality, characteristics of leg bones and immunocompetence can be important factors in these kinds of studies. It is not clear whether the level of vitamin E in meat of broilers is sufficient for stability of meat quality after being slaughtered and long storage in freezer. Because lipid oxidation is a major cause of meat quality deterioration and products of autoxidation of unsaturated fatty acids affect wholesomeness and nutritional value.^4^ Vitamin E is an important part of the body’s intracellular defense against the adverse effects of reactive oxygen and free radicals that initiate oxidation of unsaturated phospholipids.^5^

In these studies there were no reports about effect of withdrawal or reduction of vitamin supplements on the characteristics of leg bones. Because vitamin D is a calcitropic hormone involved in Ca absorption in the intestine, it is widely used as a feed supplement.^6^ Besides vitamin D, vitamins B_6_, C and K are integral factors to bone health because of their involvement in the synthesis of matrix constituents, such as collagen and osteocalcin, and formation of collagen crosslinks.^7^

Therefore this study was carried out to evaluate the effects of reduction or withdrawal of the VP from broiler diets based on corn-soybean meal during the finisher (29-43 days) period on performance, immunocompetence, characteristics of leg bones and meat quality in floor raising systems.

## Materials and Methods

**General procedure.** This study was conducted according to the University Animal Welfare Norms. Birds and housing: The average initial body weight of chicks in each pen was 42.00 ± 2.00 gram. Room temperature was kept at 34 ˚C during the first 3 days of the trial and then was reduced gradually according to age until reaching 22 ˚C at 21 day. The light was continuous during the first three days, and then the lighting regimen was 23 hr per day.

Chickens were raised until 29 day of age and fed on commercial starter and grower diets that met their nutrient requirements (Ross 308, 2007; Table 1), as described in the general procedure, weighed (1125.00 ± 9.60 g) and distributed at random into pens with five treatments with nine repetitions per treatment and 20 birds per floor pen replicate. The dietary treatments were: T1) the basal diet without VP during 29-43 days; T2) the basal diet 33% VP during 29-43 days (0.08 g kg^-1^); T3) the basal diet 66% VP during 29-43 days (1.60 g kg^-1^); T4) the basal diet 100% VP during 29-43 days (2.50 g kg^-1^) and T5) the basal diet 133% VP during 29-43 days (3.30 g kg^-1^). The ingredient composition of the experimental diet and the nutrient composition are shown in Table 1. Mash feed and water were available for *ad-libitum* consumption. Prior to formulation, all major dietary ingredients were analyzed for apparent metabolizable energy (AME_n_), amino acid (AA) profiles (according to prediction formula existing in NRC), crude protein (CP), crude fiber (CF) and ether extract (EE) contents as described by others.^8^

**Production performance.** Mortality during 29^th^ to 43^th^ days was determined for each pen. Body weight gain (BWG) and feed intake (FI) of chickens were determined at 33^th^, 38^th^ and 43^th^ days of age, then feed conversion ratio was calculated from these data. 

**Lymphoid organs weight.** At 33^th^, 38^th^ and 43^th^ days of age, birds were weighed, and then two birds from each replicate, were selected at initial body weights were similar in replicate, subjected to 6 hour fasting, reweighed, then slaughtered. The relative weight of the lymphoid organs (bursa of fabricius and spleen) was measured to the nearest 0.01 gram.

**Immunocompetence response**

**Humoral immune response.** The sheep red blood cells (SRBC) were used as T-dependent antigens to quantify the antibody response. In the trial, four birds were selected from each of the replicated groups (36/treatment) and were injected intravenously with SRBC (1% suspension in phosphate-buffered saline (PBS), 0.1 mL per chick) at 29 day of age followed by a booster injection of SRBC suspension at 7^th^ day after the first injection. Blood samples were collected at 6^th^ day after the first injection and again at 7^th^ day post booster. The serum from each sample was collected; heat inactivated at 56 ˚C for 30 min and then analyzed for total anti-SRBC antibodies as previously described.^9^ Briefly, 50 μL of serum was added in an equal amount of PBS (pH 7.6) in the first column of a 96-well V-shaped bottom plate (Corning Glass, Corning, NY, USA), and the solution was incubated for 30 min at 37 ˚C. A serial dilution was made (1:2), and 50 μL of 2% SRBC suspension was added to each well. Total antibody titers were then read after 30 min of incubation at 37 ˚C. The well immediately preceding a well with a distinct SRBC button was considered as the endpoint titer for agglutination. For MER-IgG response, 25 µL of 0.02 molar mercaptoethanol in PBS was used instead of PBS alone, followed by the pervious mentioned procedure. The difference between the total and IgG response was considered to be equal to the IgM antibody level.^10^

**DNCB challenge.** On the 34^th^ and 42^th^ days of ages 1-chloro-2, 4-dinitrobenzene (DNCB, Merck, Darmstadt, Germany) solution (10 mg mL^-1^) was spread and maintained over a 10 cm^2^ area of featherless skin (0.25 mL) on the right side of the two birds per pen. Similar position on the left side of the bird was treated by the solvent alone (acetone: olive oil, 4:1 v/v) to correct the solvent effect. The second treatment by DNCB solution (1 mg mL^-1^) was applied 34 and 42 days of ages.^11^ The skin swelling was calculated as the difference between the thickness of the skin before and after DNCB treatment was measured using a digital caliper (Mitutoyo, Tokyo, Japan).

**PHA-M induced lymphoproliferation.** Phytohem-agglutinin-M (Gibco, Grand Island, NY, USA), T-cell mitogen was injected (100 mg dissolved in 100 mL of sterile PBS) to the right toe web of two birds experiment group at 34 and 42 days of ages. The increase in toe web thickness was measured 24 hr after injection.^12^

**Thiobarbituric acid reactive substances value. **The two thigh from each replicate in 3 slaughtered ones (33, 38 and 43 days) were stored in separate oxygen permeable plastic bags at -80 ˚C until required for chemical analyses on day 90. The extent of lipid oxidation in thighs was assessed by measuring thiobarbituric acid reactive substances (TBARS) according to the method described by Cortinas *et al.* using third derivative spectrophotometry.^13^ The height of the third-order derivative peak that appeared at approximately 521.5 nm was used for calculation of malondialdehyde (MDA) concentration in the samples. Tetraethoxypropane (Sigma Chemical Co., St. Louis, Mo, USA) was used as an MDA precursor in the standard curve. TBARS was expressed as micrograms of MDA per kilogram of sample.

**Evaluation of** **bone parameters** **. **At days 33, 38 and 42 of ages, two bird of each replicate were slaughtered and bone parameters were measured. A middle toe of the right leg was used for the determination of ash, calcium and phosphorus.^8^

**Statistical analysis** ***. ***The data were subjected to ANOVA as a completely randomized design using the GLM (General Linear Model) procedure of SAS software. Anti-SRBC titers and the weight data of lymphoid organs were transformed to log_2_ and arcsine, respectively. Means were separated by Duncan’s multiple range test at significance level of *p *< 0.05. 

**Table 1:** Composition of the starter and grower diets used in the pre-experimental period

**Ingredient** **s**	**Starter diet ** **(g kg** ^-1^ **)**	**Grower diet ** **(g kg** ^-1^ **)**	**Finisher diet ** **(g kg** ^-1^ **)**
Corn	600.00	664.00	670.20
Soybean meal (440 g kg^-1^ CP) 318.80		283.00	265.90
Gluten meal	18.00	15.00	-
Soybean oil	20.00	5.00	26.80
Oyster shell	13.00	13.00	8.20
Dicalcium phosphate	20.00	12.00	18.50
Vitamin premix^1^	2.50	2.50	2.50
Trace mineral premix^2^	2.50	2.50	2.50
Sodium chloride	4.00	3.00	3.00
DL-Methionine	1.00	-	1.60
L-Lysine HCl	0.20	-	0.80
**Calculated compositions**			
AMEn, kcal kg^-1^	2950.00	2960.00	3000.00
Crude protein	215.00	210.00	207.00
Methionine	4.10	3.70	3.40
Methionine+Cystein	8.20	6.50	5.80
Lysine	11.50	9.10	8.50
Calcium	9.90	8.10	7.60
Available phosphorus (%)	4.70	4.10	3.70

1 Provides the following per kg of diet: 4.13 mg retinol, 60.00 µg chole-calciferol, 30.00 mg Dl-α-tocopherol, 3mg menadione, 2.20 mg thiamine, 8.00 mg riboflavin, 5.00 mg pyridoxine, 11.00 µg cyanocobalamin, 1.50 mg folic acid, 150.00 µg biotin, 25.00 mg calcium pantotenate, 65.00 mg nicotinic acid, 60.00 mg.

2 Provides the following per kg of diet: Mn (manganese sulphate), 40.00 mg Zn (zinc oxide), 0.33 mg I (potassium iodate), 80.00 mg Fe (ferrous sulphate), 8.00 mg Cu (copper sulphate), 0.15 mg Se (sodium selenite), 150.00 mg ethoxyquin.

## Results

**Performance** **. **Mortality for all groups was within the expected range and there was no significant difference in mortality of all treatments. The results from feed intake, body weight gains, and feed conversion ratio (FCR) is shown in Table 2. Reduction or withdrawal of the VP at different ages did not significantly affect FI, BWG and FCR (*p *> 0.05).

**Table 2:** Vitamin premix was reduction or withdrawn effects on growth parameters from 29^th^ to 43^th^ days

**Groups**	**29** ^th^ ** - 33** ^th^ ** day**			**34** ^th^ ** - 38** ^th^ ** day**			**39** ^th^ ** - 43** ^th^ ** day**		
	**FI (g)**	**BWG (g)**	**FCR (g/g)**	**FI (g)**	**BWG (g)**	**FCR (g/g)**	**FI (g)**	**BWG (g)**	**FCR (g/g)**
**T1**	117.12	71.24	1.64	135.04	79.32	1.70	160.03	84.52	1.89
**T2**	118.31	76.18	1.55	132.62	82.45	1.61	158.23	87.12	1.82
**T3**	116.78	73.26	1.59	136.76	80.36	1.70	155.89	85.65	1.82
**T4**	119.45	76.14	1.53	133.89	82.89	1.62	157.93	86.40	1.83
**T5**	117.76	73.72	1.60	137.40	80.54	1.71	160.67	85.54	1.88
**SEM**	1.23	2.41	0.09	3.51	1.76	0.05	4.65	2.90	0.07

**Table 3:** Vitamin premix reduction or withdrawal effects on Total anti-SRBC antibody, IgG, IgM titers (log HA) and IgG/IgM ratio by HA method on 34^th^ and 42^th^ days of age.

**Groups**	**Total anti-SRBC**		**IgG**		**IgM**		**IgG/IgM**	
	**34** ^th^ ** day**	**42** ^th ^ **day**	**34** ^th^ ** day**	**42** ^th^ ** day**	**34** ^th^ ** day**	**42** ^th^ ** day**	**34** ^th^ ** day**	**42** ^th^ ** day**
**T1**	3.81	4.75	2.65	3.25	1.16	1.50	2.28	2.17
**T2**	3.95	4.84	2.57	3.20	1.28	1.64	2.09	1.95
**T3**	4.00	4.96	2.71	3.33	1.29	1.63	2.10	2.04
**T4**	3.96	4.92	2.70	3.41	1.26	1.51	2.14	2.26
**T5**	3.93	4.87	2.73	3.37	1.20	1.50	2.28	2.25
**SEM**	0.11	0.12	0.09	0.11	0.08	0.08	0.10	0.16

**Table 4:** Vitamin premix reduction or withdrawal effects on lymphoid organs weight (g kg^-1^) in 33, 38 and 42 days of age

**Groups**	**33** ^th^ ** day**		**38** ^th^ ** day**		**43** ^th^ ** day**	
	**Bursa**	**Spleen**	**Bursa**	**Spleen**	**Bursa**	**Spleen**
**T1**	0.22	1.25	1.24	1.56	2.50	1.85
**T2**	0.18	1.55	1.23	1.63	2.58	2.08
**T3**	0.22	1.45	1.21	1.60	2.61	1.93
**T4**	0.19	1.30	1.15	1.76	2.78	1.89
**T5**	0.20	1.35	1.19	1.71	2.52	1.96
**SEM**	0.02	0.60	0.04	0.09	0.13	0.11

**Immunocompetence. **The effect of VP reduction or withdrawal on weights of lymphoid organs and humoral immune system response (total anti-SRBC antibody, IgG, IgM titers) are given in Tables 3 and 4. In the experiment, the humoral immune response (total anti-SRBC antibody, IgG, IgM titers) was not affected by the different treatments (*p *> 0.05). The bursa of fabricius and spleen weights were not significantly different in chickens fed on diets with various levels of VP in the experiment (*p *> 0.05). In the experiment, cellular immune response (DNCB challenge and Phytohemagglutinin-M) were not affected by different treatments at 34 and 42 days of ages (*p *> 0.05), (Table 5). 

**Table 5:** Composition of the starter and grower diets used in the pre-experimental period

**Groups**	**Increase in skin thickness (%) to PHA and DNCB at 34** ^th^ ** day**		**Increase in skin thickness (%) to PHA and DNCB at 42** ^th ^ **day**	
	**PHA**	**DNCB **	**PHA**	**DNCB **
**T 1**	0.52	1.66	0.51	0.86
**T 2**	0.55	1.62	0.54	0.83
**T 3**	0.54	1.58	0.49	0.76
**T 4**	0.48	1.55	0.50	0.72
**T 5**	0.50	1.57	0.49	0.79
**SEM**	0.07	0.06	0.02	0.04

**Bone parameters. **The effects of different levels of VP on ash, Ca and P of the bones are shown in Table 6. The results of this trial showed that VP reduction and withdrawal at 29 day of age did not impair ash, Ca or P of bones during the final period of broiler chickens (*p *> 0.05).

**Lipid oxidation in thigh meat. **Results of TBARS values showed that there were no significant differences between TBARS values of thigh samples for birds slaughtered at 33 and 38 days of age, However, TBARS values of treatment without VP were significantly higher than that of the other treatments in the birds slaughtered at 43 days of age (*p *< 0.05), (Table 7). 

**Table 6:** Vitamin premix reduction or withdrawal effects on mineral and ash content (%) of chickens in 33, 38 and 42 days of age

**Groups**	**33** ^th^ ** day**			**38** ^th^ ** day**			**43** ^th^ ** day**		
	**Ca**	**P**	**Ash**	**Ca**	**P**	**Ash**	**Ca**	**P**	**Ash**
**T1**	10.35	7.51	49.78	12.78	9.48	54.31	13.46	10.46	58.78
**T2**	11.01	8.14	50.23	12.95	9.35	55.23	14.03	9.88	59.05
**T3**	10.89	7.38	49.97	13.76	9.18	55.67	14.08	9.34	58.94
**T4**	11.23	7.47	51.20	13.08	10.61	55.87	14.52	10.95	59.34
**T5**	11.76	9.25	53.01	13.42	9.14	56.08	14.76	9.92	59.17
**SEM**	0.32	0.28	1.56	0.95	0.29	0.84	0.75	0.20	0.56

**Table 7:** Effects of VP reduction or withdrawal on the oxidative stability of broiler thighs from birds, slaughtered at 33, 38 and 42 days of age

**Groups**	**TBA** 1 ** 33** ^th^ ** day**	**TBA 38** ^th^ ** day**	**TBA 43** ^th^ ** day**
**T 1**	1.11	1.57	4.62 b
**T 2**	1.24	1.44	1.50 a
**T 3**	1.09	1.22	1.25 a
**T 4**	1.07	1.35	1.08 a
**T 5**	1.14	1.44	1.30 a
**SEM**	0.10	0.18	0.39

1 μg of malondialdehyde (MDA) per kg on a dry matter basis.

a-b Values in the same column within each experiment with different superscripts differ significantly (*p *< 0.05).

## Discussion

**Performance** **.** The findings of this study were similar to those reported by Skinner *et al.* and Maiorka *et al.* as they showed that vitamin and mineral premix withdrawal from the finisher diet of broiler chickens did not affect performance.^1^^,^^14^ Skinner *et al.* suggested that lack of a withdrawal effect could be related to the availability in the body of vitamins and minerals for further growth, as the amount of these supplements usually exceeds two or three times the recommended broiler chicken requirement in poultry diets.^14^ In contrast, omitting vitamin from the finisher diet for the same removal period decreased weight gain in three different broiler strains.^3^^,^^15^ These differences may be due to the type of rearing system (floor litter or cages) or differences in the diet composition.

It should be emphasized that the removal of VP from broiler diets does not imply that such diets are void of these essential nutrients. Unfortified diets, especially those that contain some animal protein feedstuff, may contain quantities of vitamins sufficient to meet or exceed the minimum recommended needs. Vitamin premix used in the commercial broiler industry typically provides VP at two to fourfold or more of the minimum recommended levels;^16^ thus, some storage within the carcass should be expected, especially for the fat-soluble vitamins. Under commercial growing conditions, using practical feedstuffs, it may be difficult to produce VP deficiencies in birds during the finishing period following adequate supplementation early in the growing period. Reduction of these supplements in diets fed from 29 to 43 days of age could significantly reduce growing costs with no adverse effects on performance.

**Immunocompetence. **The results of the present study are in accordance with those reported by Deyhim and Teeter and Khajali *et al.*^        2^^-^^3^ Our findings suggested that the vitamin contents of the finisher diet were sufficiently high to maintain an adequate humoral immune response or that the vitamin contents of corn and soybean meal diet were sufficiently high to compensate the lack of a VP during 29 to 43 days post-hatching. More works have been done on the effect of vitamin nutrition on immunity. The effect of vitamin E on immunocompetence of chickens is well known.^17^

In practice, nutritionists do not take into account the vitamins supplied from the natural feedstuffs. Consequently, the bird’s requirements for these nutrients are possibly met from natural feedstuffs as well as body reserves during a short-term vitamin withdrawal. It has been demonstrated that the immune system has a higher priority for circulating nutrients to maintain a humoral immune response when the grower diet is not fortified and is able to compete favorably with other tissues when nutrient levels are low.^18^

**Bone parameters. **Bone morphometric parameters have been used as indicators of bone status in nutritional and genetic research of poultry.^6^^,^^19^ There is little information about bone quality when VP was withdrawn or reduced from the diet in broilers chickens. Vitamin D has a major regulatory role in bone metabolism and bone strength.^20^ Findings of this study were similar to those reported by Christmas *et al.* as they showed that vitamin and mineral premix withdrawal from the finisher diet of broiler chickens did not affect leg abnormalities.^21^

Cantor *et al.* studied the usefulness of bone densitometry as an indicator of vitamin D status in turkey poults. The correlation coefficient between dietary vitamin D and bone mineral mass was 0.80, and between vitamin D and the percentage bone ash was 0.78.^22^ The correlation coefficient between bone mineral mass and bone ash was 0.71.^22^ They concluded that bone densitometry was as good an indicator as bone ash for the vitamin D status in turkey poults. The persistence of vitamin D in animals during periods of vitamin D deficiency may be explained by slow turnover rate of vitamin D in certain tissues, such as skin and adipose tissue. During deprivation, vitamin D in these tissues is released slowly thus meeting vitamin D needs of the animal over a longer period.^23^ Also, vitamin D can be stored in adipose tissue, liver and discharge storage time is 14 days. It seems that above reasons and access of the broilers to feces (coprophagia) in floor system can repair the vitamin D deficiency.^19^

**Lipid oxidation in thigh meat. **Vitamin E functions as a quenching agent for free radical molecules with single, highly reactive electrons in their outer shells. Highly reactive oxygen species, such as superoxide anion radical, hydroxyl radical, hydrogen peroxide, and singlet oxygen are continuously produced in the course of normal aerobic cellular metabolism. Also, phagocytic granulocytes undergo respiratory burst to produce oxygen radicals to destroy the intracellular pathogens. However, these oxidative products can, in turn, damage healthy cells if they are not eliminated. Antioxidants serve to stabilize these highly reactive free radicals, thereby maintaining the structural and functional integrity of cells.^23^^-^^24^ Therefore, anti-oxidants are very important to the health of animals. It is the anti-oxidative property of vitamin E that prevents oxidation of other lipid materials to free radicals and peroxides within cells, thus protecting the cell membrane from damage.^23^

If lipid hydroperoxides are allowed to form in the absence of adequate tocopherol levels, direct cellular tissue damage can result, in which peroxidation of lipids destroys structural integrity of the cell and causes metabolic derangements. Vitamin E reacts as a chain-breaking antioxidant, thereby neutralizing free radicals and preventing oxidation of lipids within membranes. Free radicals may not only damage their cell of origin but migrate and damage adjacent cells in which more free radicals are produced in a chain reaction leading to tissue destruction.^25^ However, it seems that withdrawal of VP did not have significant effects on broiler performance, but it can damage the tissues by decreasing the vitamin E supply for stable quality meat during time of freezing.

There are some reasons which may justify removing or reducing the usage of vitamin supplementation (an expensive essential nutrient in poultry’s diets) in finisher period diets in floor system. For instance: 1) the amount of vitamin supplements usually exceeds two or three times the recommended broiler chicken requirements in poultry diets; 2) fat-soluble vitamins may be stored by a bird in its liver and fatty tissue in sufficient quantities to meet requirements for up to 15 day or even longer; 3) there are some vitamins in diet ingredients such as wheat, barley and soybean meal that are not considered during formulation diets; 4) floor-raised broilers can access their feces to reach some vitamins which are produced in the intestine. 

In conclusion, the results of this study demonstrated that it is not possible to entirely withdraw, but that it can be possible to reduce the vitamin supplements in finisher in finisher broiler diets without negative effects on performance and meat quality during the time of freezing.
